# Personalising Antidepressant Treatment for Unipolar Depression Combining Individual Choices, Risks and big Data: The PETRUSHKA Tool: Personnalisation du traitement antidépresseur de la dépression unipolaire associant choix individuels, risques et mégadonnées: l’outil PETRUSHKA

**DOI:** 10.1177/07067437251322399

**Published:** 2025-03-13

**Authors:** Edoardo G. Ostinelli, Matt Jaquiery, Qiang Liu, Rania Elgarf, Nyla Haque, Jennifer Potts, Zhenpeng Li, Orestis Efthimiou, Sarah Markham, Roger Ede, Laurence Wainwright, Karen Barros Parron Fernandes, Bianca Barros Parron Fernandes, Paulo Victor Carpaneze Dalaqua, Anneka Tomlinson, Katharine A. Smith, Caroline Zangani, Franco De Crescenzo, Marcos Liboni, Benoit H. Mulsant, Andrea Cipriani, and on behalf of the PETRUSHKA Team

**Affiliations:** 1 Department of Psychiatry, 6396 University of Oxford, Oxford, UK; 2 Oxford Precision Psychiatry Lab, National Institute for Health Research Oxford Health Biomedical Research Centre, 6396 University of Oxford, Oxford, UK; 3 Warneford Hospital, 8955 Oxford Health NHS Foundation Trust, Oxford, UK; 4 Oxford RSE, Doctoral Training Centre, MPLS, 6396 University of Oxford, Oxford, UK; 5 School of Engineering Mathematics and Technology, Faculty of Engineering, 1980 University of Bristol, Bristol, UK; 6 545860 Institute of Primary Health Care (BIHAM), University of Bern, Bern, Switzerland; 7 Institute of Psychiatry, Psychology and Neuroscience, King's College London, London, UK; 8 School of Medicine, Pontifícia Universidade Católica do Paraná, Londrina, Paraná, Brazil; 9 Graduate Program of Health Sciences (PPGCS), Pontifícia Universidade Católica do Paraná, Curitiba, Paraná, Brazil; 10 Health Sciences Center, Londrina State University, Londrina, Paraná, Brazil; 11 Department of Psychiatry, Temerty School of Medicine, 12366 University of Toronto, Toronto, Canada; 12 Centre for Addiction and Mental Health, Toronto, Canada

**Keywords:** adult psychiatry, antidepressants, caregivers, clinical trials, depressive disorders, evidence-based medicine, pharmacotherapy

## Abstract

**Objective:**

We summarize the key steps to develop and assess an innovative online, evidence-based tool that supports shared decision-making in routine care to personalize antidepressant treatment in adults with depression. This PETRUSHKA tool is part of the PETRUSHKA trial (Personalize antidEpressant Treatment foR Unipolar depreSsion combining individual cHoices, risKs, and big datA).

**Methods:**

The PETRUSHKA tool: (a) is based on prediction models, which use a combination of advanced analytics, i.e., traditional statistics, and machine learning methods; (b) utilizes electronic health records from primary care patients with depressive disorder in England and data from randomized controlled trials on antidepressants in depression, both at aggregate and individual patient level; (c) incorporates preferences from patients and clinicians (especially about adverse events); (d) generates a ranked list of personalized treatment recommendations to inform the discussion between clinicians and patients, and facilitates the final treatment choice. The PETRUSHKA tool is implemented as a web-based application, accessible from any computer, smartphone or tablet.

**Results:**

We employed a bespoke algorithm to identify the best antidepressant for each individual patient, using patients’ clinical and demographic characteristics and harnessing the power of innovations in digital technology, large datasets and machine learning. We established a dedicated group of patient representatives that were involved in the co-production of the tool, to maximize its impact in real-world clinical practice across the world. To test the tool, we designed an international multi-site, randomized trial (target sample: 504 participants), comparing the PETRUSHKA tool with usual care to personalize pharmacological treatment in patients with depressive disorder across Brazil, Canada and the UK.

**Conclusions:**

Using evidence-based patient decision aids has been recommended to support shared decision-making when quality is assured. Future studies in precision mental health should develop multimodal web tools, incorporating patients’ preferences and their individual demographic, cultural, clinical, and genetic characteristics.

**Plain Language Summary Title:**

Tailoring antidepressant treatment to individual patients with depression: the PETRUSHKA tool

## Plain Language Summary

Antidepressants are one of the main treatments for depression. Many patients, however, are given antidepressants, which prove ineffective or cause stressful side effects for them as individuals. This happens because antidepressants are prescribed without a clear understanding of which drug is the most appropriate medication for each patient. Regulatory bodies and guidelines developers have recommended prioritizing the improvement of antidepressant treatment for depression, but this advice has not yet been translated into practice. We suggest we already have sufficient evidence to distinguish between treatments according to personal characteristics, and the preferences and values of patients themselves. People with a diagnosis of depression often need additional support during the consultation visit when they make decisions about starting a new course of treatment. By a more careful analysis of existing data, we can better tailor the choice of a specific drug to a specific person (“personalized medicine”), to increase the chances that the drug will be tolerable and effective. We developed the PETRUSHKA tool, an evidence-based online system which will help doctors and patients together choose the best antidepressant for each individual with moderate to severe symptoms of depression. For the first time, this system will bring together the best available scientific information with the preferences of patients to provide a bespoke clinical decision aid for antidepressant treatment. The PETRUSHKA tool will be tested in a scientifically sound study of depressed patients who are to be treated with antidepressants in both primary and secondary care across Brazil, Canada and the UK. During the project, patients and carers have been involved in the co-development of the PETRUSHKA tool, which provides a model that can be extended to non-pharmacological treatments and to other psychiatric and non-psychiatric disorders, such as schizophrenia, diabetes and epilepsy.

## Résumé

**Objectif:**

Nous résumons les étapes principales de l’élaboration et de l’évaluation d’un outil en ligne novateur, fondé sur des données probantes, qui soutient la prise de décision partagée dans le cadre des soins courants afin de personnaliser le traitement antidépresseur chez les adultes souffrant de dépression. L’outil PETRUSHKA fait partie de l’essai PETRUSHKA (Personalise antidEpressant Treatment foR Unipolar depreSsion combining individual cHoices, risKs and big datA).

**Méthodologie:**

L’outil PETRUSHKA : (a) est basé sur des modèles de prédiction, qui utilisent à la fois des méthodes analytiques avancées, c.-à-d. des statistiques traditionnelles, et l’apprentissage automatique; (b) utilise les dossiers médicaux électroniques de patients recevant des soins primaires et souffrant de troubles dépressifs en Angleterre, ainsi que des données provenant d’essais cliniques randomisés sur les antidépresseurs utilisés pour traiter la dépression, tant au niveau global qu’au niveau individuel; (c) intègre les préférences des patients et des cliniciens (notamment en ce qui concerne les événements indésirables); (d) génère une liste hiérarchisée de recommandations de traitement personnalisé afin de guider le dialogue entre les cliniciens et les patients et de faciliter le choix du traitement final. L’outil PETRUSHKA repose sur une application Web, accessible à partir de n’importe quel ordinateur, téléphone intelligent ou tablette.

**Résultats:**

Nous avons utilisé un algorithme sur mesure pour déterminer le meilleur antidépresseur pour chaque patient, en utilisant les caractéristiques cliniques et démographiques des patients et en exploitant la puissance des innovations relatives à la technologie numérique, aux grands jeux de données et à l’apprentissage automatique. Nous avons créé un groupe de représentants de patients qui ont été impliqués dans la coproduction de l’outil, afin de maximiser son impact dans la pratique clinique réelle partout dans le monde. Pour tester l’outil, nous avons conçu un essai randomisé international multisites (échantillon visé : 504 participants), comparant l’outil PETRUSHKA aux soins habituels pour personnaliser le traitement pharmacologique des patients souffrant de trouble dépressif au Brésil, au Canada et au Royaume-Uni.

**Conclusions:**

L’utilisation d’outils d’aide à la décision pour les patients, fondés sur des données probantes, a été recommandée pour soutenir la prise de décision partagée, lorsque la qualité est assurée. Les futures études sur la médecine de précision en santé mentale devraient servir à mettre au point des outils en ligne multimodaux, en tenant compte des préférences des patients et de leurs caractéristiques démographiques, culturelles, cliniques et génétiques.

## Background and Rationale

Depression is one of the leading causes of global burden, forecasted to increase further by 2050.^1^ There are several effective interventions for depressive disorder.^2–4^ Clinicians most frequently prescribe second-generation antidepressants for the initial treatment of depression^5^ and guidelines recommend that an adequate trial of medications should generally last 6–8 weeks before changing or stopping the antidepressant.^6,7^ However, most prescriptions are for less than 30 days.^8^ This is because, although antidepressants are relatively well tolerated and efficacious at the population level*,*^9^ a specific medication may fail to benefit or may harm an individual patient.^10^ Thus, a key challenge for clinicians and researchers is how to optimize antidepressant treatment and select the best drug for each patient.

Precision medicine is now a healthcare priority globally.^11,12^ However, mental healthcare lags behind other specialties in medicine. Despite the recent progress in precision psychiatry,^13^ the process of matching patients and treatments still relies on trial and error, delaying clinical improvement and increasing the harms and costs associated with treatment.^14^ The need for other ways of personalizing treatment has long been advocated by many organizations, including the National Institute for Health and Care Excellence in the UK.^15^ However, to date, the impact of most of these proposed alternative approaches has not been adequately evaluated. One key component of the personalization of care is shared decision-making. This is envisioned as a collaborative process that involves patients, their carers, and health providers working together to reach a joint decision about treatment.^16^

Many factors may influence the effects of an antidepressant in patients with depressive disorder, including demographic (e.g., age, gender, ethnicity), clinical (e.g., severity of symptoms, number of previous episodes, previous treatments, body mass index), environmental (e.g., co-medications, smoking, diet) and genetic variables.^17^ New approaches are needed to incorporate clinically meaningful predictors and support shared decision-making using patient decision aids,^18^ which are tools designed to facilitate the shared discussion about healthcare options between patients and clinicians. As part of PETRUSHKA (Personalizing antidEpressant TReatment for Unipolar depreSsion combining individual cHoices, risKs, and big datA),^19^ we developed an online, evidence-based patient decision aid to support shared decision-making in real-world clinical settings (PETRUSHKA tool).

## Petrushka Tool

The PETRUSHKA tool is a patient decision aid that employs a bespoke algorithm to identify which antidepressants work better for each individual patient.

The algorithm:

- Uses the individual patient-level data of people taking licensed antidepressants for depression from (i) randomized controlled trials and (ii) real-world electronic health records;^20^
- is based on several prediction models using a combination of advanced statistical and machine learning methods;^21^
- incorporates preferences from patients and clinicians elicited in real time, with a focus on common non-serious adverse events;^22,23^
- generates a personalized ranked list of recommended antidepressants that inform the discussion between providers and patients, and the final selection.

### Patient and Public Involvement

The PETRUSHKA tool is implemented in the form of a web-based application, accessible from any internet-connected device (computer, tablet, smartphone), and was coproduced with people with lived experience of depressive and other mental health conditions. Based on our previous work,^24^ in April 2020, we collected initial feedback about the clinical value and practical utility of patient decision aids from a total of 15 people with personal experience of depression, with caring responsibilities, or with interest in mental health and new technologies, recruited throughout the UK. The group initially met monthly for 6 months to:

- discuss the selection of the most relevant adverse events;
- understand patients’ expectations of the tool and how the research team can address these;
- agree how to communicate potential harms associated with antidepressants and related uncertainty.

Using a 2-h demonstration of the screens and selection mechanisms, we wanted to ascertain the patient and public involvement (PPI) approach to the ranking and visualization of the tool. Overall, the feedback from patients was positive and provided practical information on how such a tool could be developed and implemented.

### Clinician Involvement

In May 2020, we also conducted informal, semi-structured interviews with 10 clinicians from the UK, to gather initial feedback on the interface of the PETRUSHKA tool and how to use it in practice, including its integration into existing booking systems or linkage with electronic health records. Much of the discussion centred on the best ways to present information on the benefits and harms associated with antidepressants to patients. These interviews provided feedback from experienced clinicians about what they look for during a shared decision-making process, and how they normally discuss adverse events with patients and carers.

### Data Input

The data input used to build the predictive algorithms of the PETRUSHKA tool stems from two sources:

1. *Individual participant data (IPD) from double-blind randomized controlled trials (RCTs)—to estimate the relative performance between antidepressants.*

Following our previous publication,^9^ we successfully obtained IPD from 130 double-blind RCTs (about 40,000 participants) on acute treatment of depressive disorders in adults using 16 different antidepressants (agomelatine, amitriptyline, bupropion, citalopram, clomipramine, duloxetine, escitalopram, fluoxetine, fluvoxamine, imipramine, mirtazapine, paroxetine, sertraline, trazodone, venlafaxine, vortioxetine) or placebo (Table 1). After signing formal agreements, pharmaceutical companies provided pseudonymized data related to hundreds of variables (including demographic characteristics, medical and psychiatric history, allocated treatment, depression severity, medication, results of psychiatric assessments and blood tests, and adverse events) at multiple time points, either directly or via a data hosting platform. Using a subset of variables (Table 1), we analyzed these IPDs to build several predictive models considering differences between specific antidepressants in terms of efficacy (symptom severity on a validated rating scale), acceptability (discontinuation of the antidepressant due to any cause), and occurrence of specific adverse events during 8 weeks of treatment.

2. *Real-world data from electronic health records—to estimate absolute risks*.

**Table 1.** Description of the Randomized Controlled Trials (RCTs) Contributing to the Individual Patient Data Network Meta-Analysis and Covariates for the Prediction Model.

Study	Comparisons (16 ADs or placebo)	Covariates (predictors)	
130 double-blind randomized controlled trials comparing antidepressants as monotherapy against each other or versus placebo in the acute treatment of major depressive disorder (bout 40,000 participants)	Agomelatine Amitriptyline Bupropion Citalopram Clomipramine Duloxetine Escitalopram Fluoxetine Fluvoxamine Imipramine Mirtazapine Paroxetine Sertraline Trazodone Venlafaxine Vortioxetine Placebo	Demographics	Clinical Information (mean ± SD)
		Age (mean: 44.8 ± 14.2) Sex (females: 25,735—64.3%)	Baseline HDRS total score (23.85 ± 3.99)
			Baseline HDRS item 3: Suicide (0.92 ± 0.86)
			Baseline HDRS item 4: Insomnia: early in the night (1.32 ± 0.80)
			Baseline HDRS item 6: Insomnia: early in the morning (1.25 ± 0.79)
			Baseline HDRS item 10: Anxiety psychic (2.25 ± 0.75)
			Baseline HDRS item 11: Anxiety somatic (1.75 ± 0.80)
			Baseline HDRS item 13: General somatic symptoms (1.65 ± 0.54)
			Baseline HDRS item 17: Insight (0.25 ± 0.48)

*Note*: The information is calculated based on 40,013 participants across 130 RCTs on 3 platforms (in-house, Vivli and SAS). The number of participants per RCT is between 10 and 970, with the median number of participants per RCT being 279. ADs: antidepressants. SD: standard deviation.

We used data from QResearch (https://www.qresearch.org/), a large, population-based dataset derived from anonymized primary care health records of over 25 million patients in England, involving longitudinal information about sociodemographic characteristics (including age, gender, ethnicity, and socioeconomic status based on postcode), prescriptions (including dosage and duration), and clinical outcomes (including severity of symptoms) at an individual level. Starting from over 1.5 million patients with depressive disorders, we selected 187,757 patients who were taking fluoxetine, as fluoxetine was the most prescribed antidepressant and was chosen as the reference antidepressant in our analysis (Supplementary Material, Figure 1). These patients provided information about the predictors (such as sociodemographic characteristics, depression details, comorbidities, and use of other medications at baseline) and were finally included in the analysis of efficacy and acceptability after 8 weeks of treatment.^25^ Unfortunately, the reporting of adverse events in QResearch was neither accurate nor comprehensive, so we did not include this information in our analyses.

3. *Incorporating patients’ preferences*

We used data on adverse events to elicit in real time and incorporate individual patient's preferences about the specific adverse effects they would find more bothersome than others.

### Analysis

Using RCT and QResearch datasets and employing both statistical and machine learning methods, we developed and internally validated a set of multivariable prediction models to estimate efficacy, treatment discontinuation, and occurrence of specific adverse events at the individual patient level.^26^ Predictors included sociodemographic and clinical factors (age, sex assigned at birth, ethnicity, socioeconomic status, BMI, smoking status), depression-specific variables (severity of symptoms on PHQ-9, HAMD score on item 3, 4, 6, 10, 11, 13, 17, the total score on HAMD, past use of antidepressant/SSRI/fluoxetine or psychotherapy, previous referral to secondary care, age at first diagnosis, childhood maltreatment), comorbid conditions (anxiety, chronic inflammatory diseases, coronary heart disease, diabetes, epilepsy/seizures, hypothyroidism, migraine, stroke/transient ischaemic attack), and baseline use of other medications (anticoagulants, anticonvulsants, antihypertensives, aspirin, bisphosphonates, hypnotics/anxiolytics, hormone replacement therapy, non-steroidal anti-inflammatory drugs, oral contraceptives, statins). Missing data were imputed via a multiple imputation approach of the entire dataset, using additive regressions to impute values when actual values were not available.^27^ Imputations were performed separately for each source of data, i.e., RCTs (we used the “mice” package in R) and QResearch.

**Efficacy: **Our first step was to develop a model to predict the symptom severity after 8 weeks based on patients in the QResearch dataset who were taking fluoxetine. Our starting point was a ridge regression model, in which we accounted for non-linear effects between the outcome and the continuous predictors using restricted cubic splines, assuming four knots. We also developed a machine learning model, i.e. a multi-layer perceptron deep neural network with three hidden layers and 256 neurons per layer. Additionally, we developed a meta-learner model, which combined the results from both statistical and machine learning models.^24^ The meta-learner was based on a multilayer perceptron neural network structure and aimed to harnesses the capabilities of both approaches and combine their contributions to maximize the predictive performance. Each of the 10 imputed datasets was analysed separately, and the results were combined. After developing the prediction model, we assessed its predictive performance. To avoid overfitting, the performance of the model was quantified in an internal 10k-fold cross-validation using mean squared absolute error. The mean absolute error of the meta-learner in cross-validation was 4.56. To harmonise the observational data with the RCTs, the Patient Health Questionnaire–9 (PHQ-9) scores reported in QResearch were converted into total scores on the Hamilton Depression Rating Scale (HDRS) using a validated method.^28,29^ We prioritised HDRS over other rating scales for depression, because most RCTs in our dataset used HDRS to assess the severity of symptoms. The resulted model was capable of predicting symptoms severity (i.e., HDRS scores) after 8 weeks of fluoxetine treatment. Using the RCT data, we then carried out an IPD random effects network meta-analysis (IPD-NMA) to calculate the relative effects of other antidepressants versus fluoxetine based on HDRS scores.^30^ The model included several baseline variables considered to be potential modifiers (e.g., severity of symptoms at baseline, age, gender and specific HDRS items). The output of this second step of the analysis was a model that can predict patient-level comparative treatment effects (difference in HDRS) between fluoxetine and any other antidepressant, given baseline patient covariates. Finally, we combined the two models to predict outcomes at the individual patient level for any antidepressant: “predicted outcome with antidepressant X” = “predicted absolute effect with fluoxetine (using the model developed using the QResearch dataset) + relative effects of X vs fluoxetine (using the model developed using IPD from RCTs)” **Αll-cause treatment discontinuation: **We first developed a prediction model using the QResearch dataset to predict the probability of stopping the antidepressant after 8 weeks in patients who were taking fluoxetine. We used the same baseline predictors as in the efficacy analysis. Internal validation was performed as above, and we assessed both discrimination measures, i.e. the area under the receiver operating characteristic curve (AUC), and the calibration slope. Next, we used the aggregated data from the GRISELDA dataset89 and performed a random effects NMA to estimate odds ratios for each antidepressant versus fluoxetine.^31^ Then, for each patient we combined the absolute probability of discontinuing fluoxetine given the patient’s characteristics (obtained from the analysis of the QResearch dataset) with the odds ratios (obtained from the analysis of the RCTs dataset) to obtain the absolute probabilities of discontinuing any specific antidepressant. **Adverse events: **Using IPD from RCTs, we first selected the 30 most common adverse events (abnormal dreams, agitation, anxiety, cold symptoms, constipation, decreased appetite, diarrhoea, dizziness, dry mouth, erectile disorder, fatigue, headache, hypertension, hypotension, infections, insomnia, nausea, pain, palpitations, respiratory disorder, sexual dysfunction, sleepiness, sore stomach, stomach pain, sweating, tremor, vision disorder, vomiting, weight gain, weight loss)^22^ and then did a minimum sample size calculation.^32^ A total of 12 adverse events met the minimum sample size requirements. We selected adverse events based on sample size (informed by the number of patients providing data for the paroxetine arm, n = 5720); we further excluded adverse events if the AUC of the corresponding model was less than 0.55. For each of them, we developed a ridge logistic regression model to predict their absolute probability in association with the reference antidepressant (i.e. the antidepressant with the largest sample in the RCT dataset, namely paroxetine). Next, we performed an NMA of aggregate data from GRISELDA9 to calculate odds ratios for all other antidepressants vs paroxetine. Finally, we used the same strategy as for the all-cause treatment discontinuation to combine the two models and obtain the probability for each adverse event and for each antidepressant given patient-level baseline characteristics.

#### Software

Analyses were performed in Python and R, using the following packages: mice, netmeta, and glmnet (full codes and syntax are available from the authors, upon request).

### Web-Based Platform

**Figure 1.**
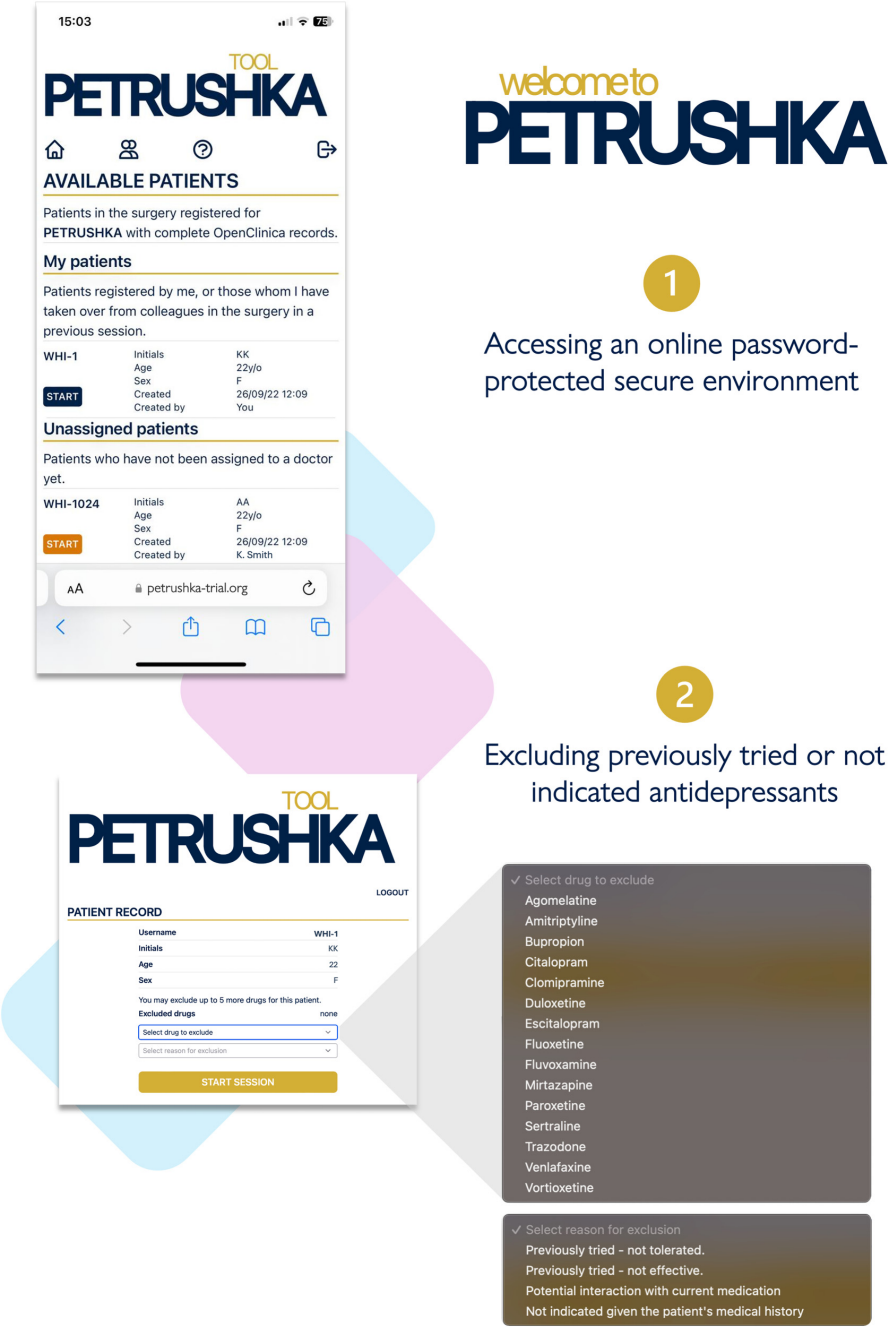
Model pipeline for the backend of the algorithm (PETRUSHKA tool)—see text for full details.

*Note*: GRISELDA = network meta-analysis of aggregate data about antidepressants in depression (Cipriani et al., Lancet 2018); HAMD = Hamilton Depression Rating Scale; IPD-NMA = individual patient data network meta-analysis; MCDA = multiple-criteria decision analysis; AQ12 PHQ-9 = Patient Health Questionnaire, 9 items; RCT = randomized controlled trial.

A web-based platform for the PETRUSHKA tool was developed using Django, a Python-based free and open-source framework. A web-based platform for the PETRUSHKA tool was developed using Django, a Python-based free and open-source framework. We considered the three outcomes (efficacy, all-cause treatment discontinuation and adverse events) as independent, and jointly assessed them following a multiple-criteria decision analysis framework using a partial value function (Figure 1).

- Efficacy: We developed a meta-learner to predict the PHQ9 absolute score of fluoxetine using the QResearch dataset and converted the predicted scores to the HAMD score. We developed an IPD-NMA model to predict HAMD relative scores of all drugs on the IPD RCT data on 3 platforms. We then obtained absolute scores of all drugs using fluoxetine absolute score and relative scores of all drugs.
- All-cause treatment discontinuation (dropout rate): We developed a meta-learner to predict the dropout absolute probability of an event on fluoxetine using the QResearch dataset. We developed an aggregate data NMA (AD-NMA) model to predict relative probabilities of dropout using the GRISELDA dataset.^9^ We then obtained absolute probabilities of all drugs using fluoxetine dropout absolute probability and dropout relative probabilities of all drugs.
- Adverse events (AEs): For the AEs that met the sample size and the area under the curve (AUC) requirements, we developed ridge logistic regression models to predict the AE absolute probabilities of paroxetine using the in-house RCT dataset. For the AEs that did not meet the sample size and AUC requirements, we developed prediction models to estimate AE absolute probabilities using the GRISELDA dataset.^9^ For all 30 AEs, we then developed AD-NMA models to predict AE relative probabilities using the GRISELDA dataset.^9^
- Overall recommendation value: We considered outcomes (efficacy, all-cause treatment discontinuation and adverse events) as independent and jointly assessed them following a multiple-criteria decision analysis (MCDA) framework using a partial value function to generate a final recommendation value ranging from 0 to 1. For the purposes of the first iteration of the PETRUSHKA tool, “efficacy”, “acceptability” and “side effect” received overall equal weights (i.e., 0.333). We then distributed the weight of “side effect” as a category across the different specific adverse events based on the preferences elicited in real time from individual participants.

The hosting and related application programming interface (APIs) were provided by Amazon Web Services (AWS). The platform accepts inputs (e.g., patient or provider preferences) via a web-based interface (front end) and data previously collected in another platform, called OpenClinica (i.e., demographics and questionnaires), via a dedicated API in Sentry. Data are then transferred to the back end, the environment where the prediction models sit. The output of the prediction models (i.e., specific prediction for each patient) is then transferred to the front-end layer, where it is communicated to patients and clinicians. No clinical data are stored within the platform, and all the meta-data resulting from human–computer interaction is then transferred back to Sentry. Access to the platform is password-protected via user-specific login. Clinicians are provided with individual login credentials, while a temporary login is generated for patients, as they need to use the PETRUSHKA tool only once when starting a new antidepressant. Patients and providers can go through the assessment together in real-time on separate devices, allowing for remote consultations to use the tool just as easily as in-person ones.

In parallel to the development pipeline, we worked alongside the PPI group to ensure patients would be able to use and understand the tool. A series of hypothetical scenarios about real-world clinical settings and simulation sessions were provided via dedicated meetings, which focused on:

- key features to be used throughout the platform;
- communication of benefit-harm information to support the shared decision-making process;
- additional feedback on available or desired features (e.g., elicitation of individual preferences as optional to account for people not willing to have an active role in the shared decision-making process).

### How the Tool is Being Tested

We designed an international multi-site, two-arm, randomized, superiority trial comparing the PETRUSHKA tool with usual care to personalize pharmacological treatment in patients with depressive disorder. Funded by the UK National Institute for Health and Care Research (NCT05608330, https://clinicaltrials.gov/study/NCT05608330?term = NCT05608330&rank = 1), the study aimed to recruit 504 participants from: multiple sites across primary and secondary care in England (GP practices and mental health NHS Trusts); one site in Canada (the Centre for Addiction and Mental Health in Toronto); and two sites in Brazil (the Pontifícia Universidade Católica do Paraná, Londrina Campus and the Universidade Estadual de Londrina). The total duration of the follow-up was 24 weeks, with the primary outcome assessed at 8 weeks. Participants were aged 18–74 years (inclusive) with a diagnosis of a non-bipolar, non-psychotic depressive disorder, who required and were willing to start treatment with antidepressant monotherapy.

The main objective of the trial was to determine whether using the PETRUSHKA tool to identify the recommended antidepressant at baseline was associated after 8 weeks with more people taking the same antidepressant than with usual care. Usual care (or treatment as usual) refers to the routine standard care delivered by providers, where the selection of the oral antidepressant was based on their clinical judgement and experience, or clinical guidelines. Patients with a depressive disorder were considered for the study only after the patient and clinician jointly agreed on starting an antidepressant treatment for the depressive disorder. Most of the recruitment was expected to be in primary care as this is where patients with depressive disorder are usually seen, especially in the UK. However, patients from secondary care were also included to increase the generalizability of the findings. Based on each site and local health advisory board's preference and licensing status, the list of antidepressants available for prescription (i.e., sitting in the backend) was modified and adapted to match the list of drugs allowed locally.

### Eliciting Preferences

**Figure 2.**
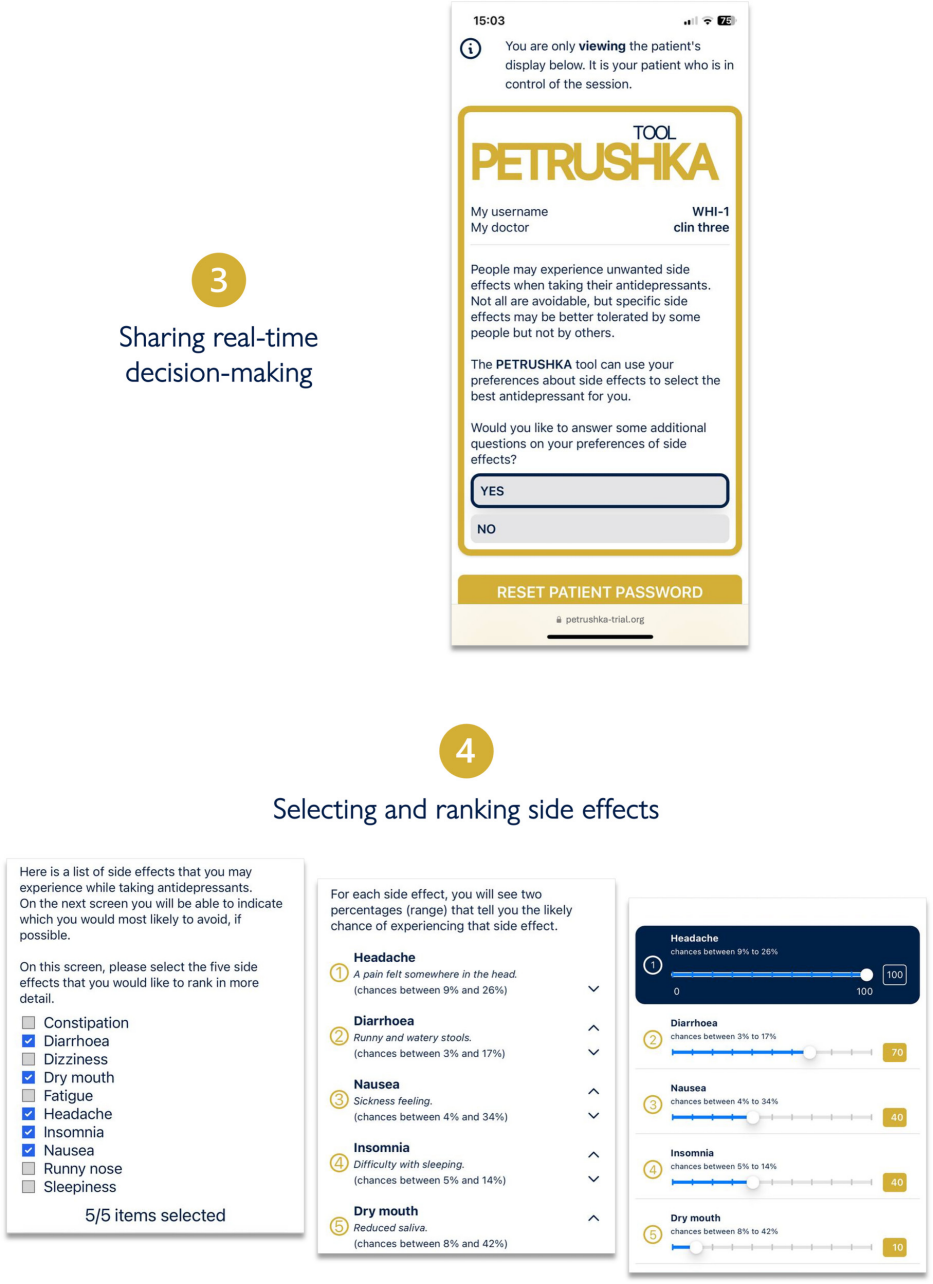
Clinicians can log in to the PETRUSHKA tool web app, accessing patients from their clinic, either registered by themselves or with their colleagues. After the correct patient is selected, clinicians can optionally exclude some antidepressants based on the patient's medical history and ongoing medications.

**Figure 3.**
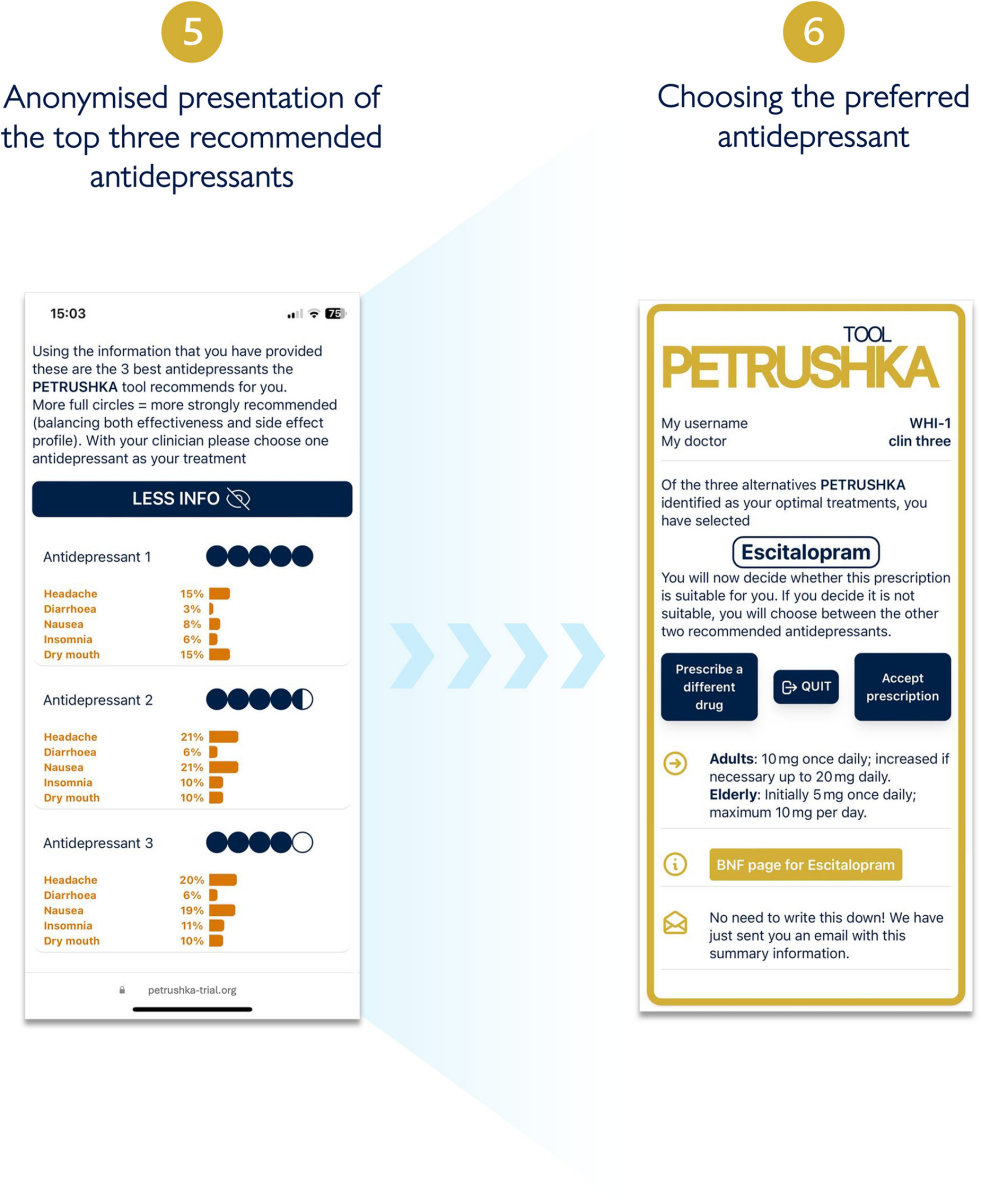
The clinician and the patient will simultaneously access the PETRUSHKA tool from any internet-connected device. In real-time, the patient will be in charge of answering several questions: whether they would like to provide additional information on their preferences about side effects and which side effects are more important for them to avoid.

**Figure 4.**
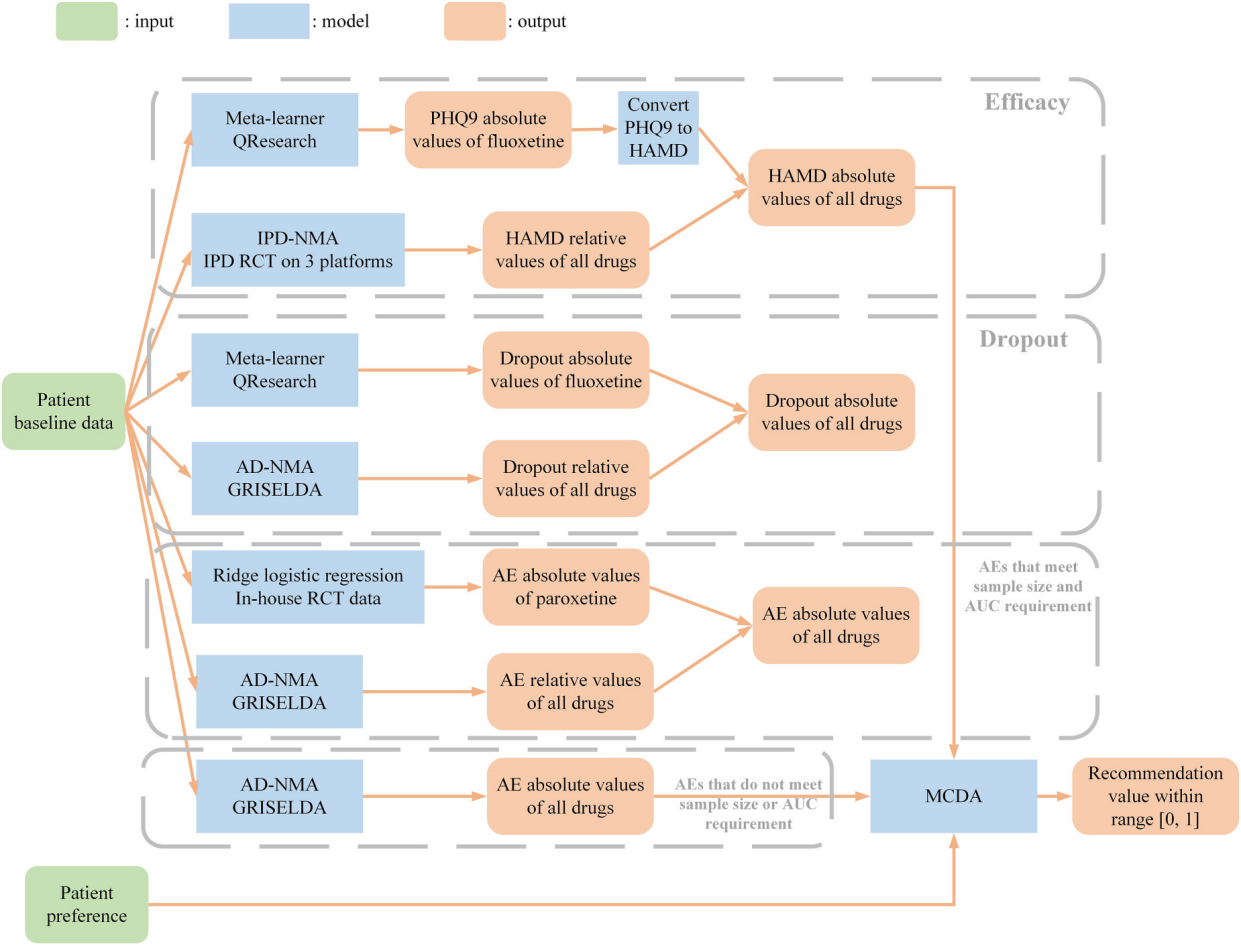
Three antidepressants are selected by the PETRUSHKA tool based on the patient individual characteristics and their preferences on side effects (if available). The five blue dots visually represent how strongly the PETRUSHKA tool is suggesting a specific antidepressant, jointly taking into consideration efficacy, acceptability, and the selected side effects. A breakdown of the selected five side effects for the three antidepressants is available both in numbers and depicted as bars. To avoid information flooding, predictions on all the 30 adverse events are available exclusively on the clinician screen, as additional information. Once an antidepressant is selected, the name is disclosed together with additional information on how to start it.

After participants provided informed consent, the data input into the web-based PETRUSHKA tool was at the point of care and came from two sources: patient and clinician (Figure 2). By use of questionnaires and validated rating scales (in English or in Portuguese, as appropriate), each patient was asked to provide the demographic (e.g., age, gender, ethnicity, etc.) and clinical characteristics (severity of symptoms, previous antidepressant treatments, etc.) that were necessary to run the prediction models and the treatment algorithm. Participants were asked a series of questions probing their feelings and preferences about adverse effects (Figure 3). During the same consultation, clinicians were asked to input any clinically relevant data, including their clinical evaluation, concomitant medications, or comorbid physical conditions (e.g., heart problems or other clinical contraindications). Using all this information, the algorithm generated a ranking list of the three top antidepressants, with visuals to show how strongly each treatment was recommended and with numbers to illustrate the associated predicted chance of having specific adverse effects (Figure 4). These personalized treatment recommendations informed the shared decision-making process between providers and patients, which produced the final treatment decision that was recorded in OpenClinica.

## Discussion

In this paper, we summarized the key steps that we followed to develop and assess the PETRUSHKA tool, an innovative tool that supports shared decision-making for the treatment of depressive disorders in real-world clinical settings.^19^

Using patient decision aids has been recommended by the National Institute for Health and Care Excellence (NICE) to support shared decision-making if the quality is assured, if it reflects the evidence-based best practice, and if it is relevant to the decision that needs to be made and the specific clinical setting.^16^ The PETRUSHKA tool has many strengths: it is evidence-based; it is co-developed with patients and clinicians; it incorporates patients’ preferences guiding the clinical discussion of harms and benefits in the context of each person's life and what matters to them, particularly in terms of adverse events (patient-centred care); it supports probabilistic decision-making using a combination of numbers and graphical presentations, such as pictographs, favouring absolute over relative risks and avoiding statistical jargon to facilitate and enhance understanding by patients and clinicians;^33,34^ it is available in English and Brazilian Portuguese so can be used internationally and in different and diverse cultural settings.^35^

Recently, other online websites (Depression Medication Choice Decision Aid, https://depressiondecisionaid.mayoclinic.org/) or digital tools (Psymatik, https://www.psymatik.com/) have been developed to support care delivery and facilitate more collaborative and evidence-based prescribing decisions. Some of these tools use a comprehensive dataset of side effects from antidepressant and antipsychotic medications and allow for personalization according to individual preferences. However, the development of these tools only used data from randomized controlled trials (i.e., populations that might not wholly represent real-world patients) and did not account for patient characteristics such as age, race and ethnicity, and sex, which might impact the risks of side effects.^36^

For the PETRUSHKA project, we set up a dedicated PPI group across the UK, that worked with us regularly at each stage of the tool's co-design, from inception to invention and development. The ability and opportunity to engage and proceed with the PPI group were significantly delayed and hindered by the COVID-19 pandemic, but these delays have enabled us to bring together a more diverse and engaged group because of the virtual setting used for all our meetings: remote technology has helped to recruit people of diverse background and socio-economic status from across the country. Developing a common language of adverse events, accessible to patients and clinicians, is a critical step in moving towards shared decision-making that is patient-centred.^37^ Such a tool could improve transparency, communication, and alignment of research output and goals among patients, clinicians, and researchers. Although resources on the language and definitions of adverse effects of antidepressants exist, there is little evidence of collaboration with people with lived experience to maximize their usefulness, acceptability, and accessibility. To fill this gap, and in line with the work done so far on the PETRUSHKA project, we co-developed a freely accessible, patient-friendly dictionary of potential harms associated with antidepressants to help patients express meaningful preferences about specific adverse effects.^38^ This online dictionary demystifies obscure and opaque terminology for patients and is an effective tool to access information about potential harms and make shared decisions in routine care (https://thesymptomglossary.com).

The PETRUSHKA tool has some important limitations: (1) the treatment options are limited to antidepressant monotherapy and augmentation/adjunctive treatments are not covered, so the recommendations from the tool apply only to people with depression who may benefit from a first- or second-line treatment (i.e., patients with treatment-resistant depression are not eligible); some antidepressants are not included in the algorithm (e.g., desvenlafaxine, levomilnacipran, nortriptyline) because we managed only to have access to a subset of all marketed antidepressants (we contacted pharmaceutical companies, but they did not agree to share the IPD from their RCTs); the tool addresses a large number but not the full list of common adverse effects, and several side-effects that are significant for patients (e.g., emotional blunting and mental pain) have not been included in our analyses.

In the current version of the PETRUSHKA tool, the prediction models included only tabular data on sociodemographic and clinical predictors, as this was a pragmatic approach to the challenges of precision mental health (pragmatic precision mental health).^39^ In future versions of the tool, other predictors could be added. For example, updated guidelines and recent pharmacogenetic studies in depression have shown that taking into account genotypes and drug metabolism enzymes can improve patients’ responses and reduce the occurrence of adverse events and treatment discontinuation.^40–43^ Notably, pharmacogenetic variants have been studied extensively across global populations; they have similar influences, but their frequencies often differ between ancestry groups, e.g., 3% (Europeans), 7% (Africans), 17% (South Asians), and 42% (East Asians) have the CYP2D6*10 haplotype.^44^ Advanced and economical DNA sequencing technologies are now available, making them suitable for use in low-resource environments and returning results in less than 48 h. Hence, pharmacogenomic variants and polygenic risk scores could provide timely individual-level information for further personalizing treatments.

Future studies in precision mental health should develop and test an evidence-based multimodal web tool integrating new information as it becomes available (such as pharmacogenetic factors discussed earlier). This will help patients and clinicians choose the best pharmacotherapy for depressive disorders together, based on patient’s preferences and their individual demographic, cultural, clinical, and genetic profiles. This precision psychiatry approach should be used in other fields of mental health, especially where there are unmet needs, and the use of digital technology can materially improve the outcomes of patients globally.^45^

## Supplemental Material

sj-docx-1-cpa-10.1177_07067437251322399 - Supplemental material for Personalising Antidepressant Treatment for Unipolar Depression Combining Individual Choices, Risks and big Data: The PETRUSHKA Tool: Personnalisation du traitement antidépresseur de la dépression unipolaire associant choix individuels, risques et mégadonnées: l’outil PETRUSHKA Supplemental material, sj-docx-1-cpa-10.1177_07067437251322399 for Personalising Antidepressant Treatment for Unipolar Depression Combining Individual Choices, Risks and big Data: The PETRUSHKA Tool: Personnalisation du traitement antidépresseur de la dépression unipolaire associant choix individuels, risques et mégadonnées: l’outil PETRUSHKA by Edoardo G. Ostinelli, Matt Jaquiery, Qiang Liu, Rania Elgarf, Nyla Haque, Jennifer Potts, Zhenpeng Li, Orestis Efthimiou, Sarah Markham, Roger Ede, Laurence Wainwright, Karen Barros Parron Fernandes, Bianca Barros Parron Fernandes, Paulo Victor Carpaneze Dalaqua, Anneka Tomlinson, Katharine A. Smith, Caroline Zangani, Franco De Crescenzo, Marcos Liboni, Benoit H. Mulsant, Andrea Cipriani and in The Canadian Journal of Psychiatry
