# Supplementary material for: Personalising Antidepressant Treatment for Unipolar Depression Combining Individual Choices, Risks and big Data: The PETRUSHKA Tool: Personnalisation du traitement antidépresseur de la dépression unipolaire associant choix individuels, risques et mégadonnées: l’outil PETRUSHKA
Source: Can J Psychiatry. 2025 Mar 13;70(10):768–81. doi: 10.1177/07067437251322399 (PMC11907562; doi:10.1177/07067437251322399)
Supplement: sj-docx-1-cpa-10.1177_07067437251322399 - Supplemental material for Personalising Antidepressant Treatment for Unipolar Depression Combining Individual Choices, Risks and big Data: The PETRUSHKA Tool: Personnalisation du traitement antidépresseur de la dépression unipolaire associant choix individue [file sj-docx-1-cpa-10.1177_07067437251322399.docx]

**Personalising antidepressant treatment for unipolar depression combining individual choices, risks and big data: the PETRUSHKA tool**

Edoardo G Ostinelli,^1-3^ Matt Jaquiery,^4^ Qiang Liu,^1,2,5^ Rania Elgarf,^1,2^ Nyla Haque,^1,2^ Jennifer Potts,^1,2^ Zhenpeng Li,^1,2^ Orestis Efthimiou,^6^ Sarah Markham,^7^ Roger Ede,^3^ Laurence Wainwright,^1^ Karen Barros Parron Fernandes,^8,9^ Bianca Barros Parron Fernandes,^8^ Paulo Victor Carpaneze Dalaqua,^8^ Anneka Tomlinson,^1-3^ Katharine A Smith, ^1-3^ Caroline Zangani, ^1-3^ Franco De Crescenzo,^1,2^ Marcos Liboni,^10^ Benoit H Mulsant,^11,12^ Andrea Cipriani ^1-3^ on behalf of the PETRUSHKA Team

**SUPPLEMENTARY MATERIAL**

**Analysis**

*Efficacy*

Our first step was to develop a model to predict the symptom severity after 8 weeks based on patients in the QResearch dataset who were taking fluoxetine. Our starting point was a ridge regression model, in which we accounted for non-linear effects between the outcome and the continuous predictors using restricted cubic splines, assuming four knots. We also developed a machine learning model, i.e. a multi-layer perceptron deep neural network with three hidden layers and 256 neurons per layer. Additionally, we developed a meta-learner model, which combined the results from both statistical and machine learning models.^24^ The meta-learner was based on a multilayer perceptron neural network structure and aimed to harnesses the capabilities of both approaches and combine their contributions to maximize the predictive performance. Each of the 10 imputed datasets was analysed separately, and the results were combined. After developing the prediction model, we assessed its predictive performance. To avoid overfitting, the performance of the model was quantified in an internal 10-fold cross-validation using mean absolute error. The mean absolute error of the meta-learner in cross-validation was 4.56. To harmonise the observational data with the RCTs, the Patient Health Questionnaire–9 (PHQ-9) scores reported in QResearch were converted into total scores on the Hamilton Depression Rating Scale (HDRS) using a validated method.^28,29^ We prioritised HDRS over other rating scales for depression, because most RCTs in our dataset used HDRS to assess the severity of symptoms. The resulted model was capable of predicting symptoms severity (i.e., HDRS scores) after 8 weeks of fluoxetine treatment.

Using the RCT data, we then carried out an IPD random effects network meta-analysis (IPD-NMA) to calculate the relative effects of other antidepressants versus fluoxetine based on HDRS scores.^30^ The model included several baseline variables considered to be potential modifiers (e.g., severity of symptoms at baseline, age, gender and specific HDRS items). The output of this second step of the analysis was a model that can predict patient-level comparative treatment effects (difference in HDRS) between fluoxetine and any other antidepressant, given baseline patient covariates.

Finally, we combined the two models to predict outcomes at the individual patient level for any antidepressant: “*predicted outcome with antidepressant X” = “predicted absolute effect with fluoxetine (using the model developed using the QResearch dataset) + relative effects of X vs fluoxetine (using the model developed using IPD from RCTs)”*

*Αll-cause treatment discontinuation*

We first developed a prediction model using the QResearch dataset to predict the probability of stopping the antidepressant after 8 weeks in patients who were taking fluoxetine. We used the same baseline predictors as in the efficacy analysis. Internal validation was performed as above, and we assessed both discrimination measures, i.e. the area under the receiver operating characteristic curve (AUC), and the calibration slope. Next, we used the aggregated data from the GRISELDA dataset^9^ and performed a random effects NMA to estimate odds ratios for each antidepressant versus fluoxetine.^31^ Then, for each patient we combined the absolute probability of discontinuing fluoxetine given the patient’s characteristics (obtained from the analysis of the QResearch dataset) with the odds ratios (obtained from the analysis of the RCTs dataset) to obtain the absolute probabilities of discontinuing any specific antidepressant.

*Adverse events*

Using IPD from RCTs, we first selected the 30 most common adverse events (abnormal dreams, agitation, anxiety, cold symptoms, constipation, decreased appetite, diarrhoea, dizziness, dry mouth, erectile disorder, fatigue, headache, hypertension, hypotension, infections, insomnia, nausea, pain, palpitations, respiratory disorder, sexual dysfunction, sleepiness, sore stomach, stomach pain, sweating, tremor, vision disorder, vomiting, weight gain, weight loss)^22^ and then did a minimum sample size calculation.^32^ A total of 12 adverse events met the minimum sample size requirements. We selected adverse events based on sample size (informed by the number of patients providing data for the paroxetine arm, n = 5720); we further excluded adverse events if the AUC of the corresponding model was less than 0.55. For each of them, we developed a ridge logistic regression model to predict their absolute probability in association with the reference antidepressant (i.e. the antidepressant with the largest sample in the RCT dataset, namely paroxetine). Next, we performed a NMA of aggregate data from GRISELDA^9^ to calculate odds ratios for all other antidepressants vs paroxetine. Finally, we used the same strategy as for the all-cause treatment discontinuation to combine the two models and obtain the probability for each adverse event and for each antidepressant given patient-level baseline characteristics.


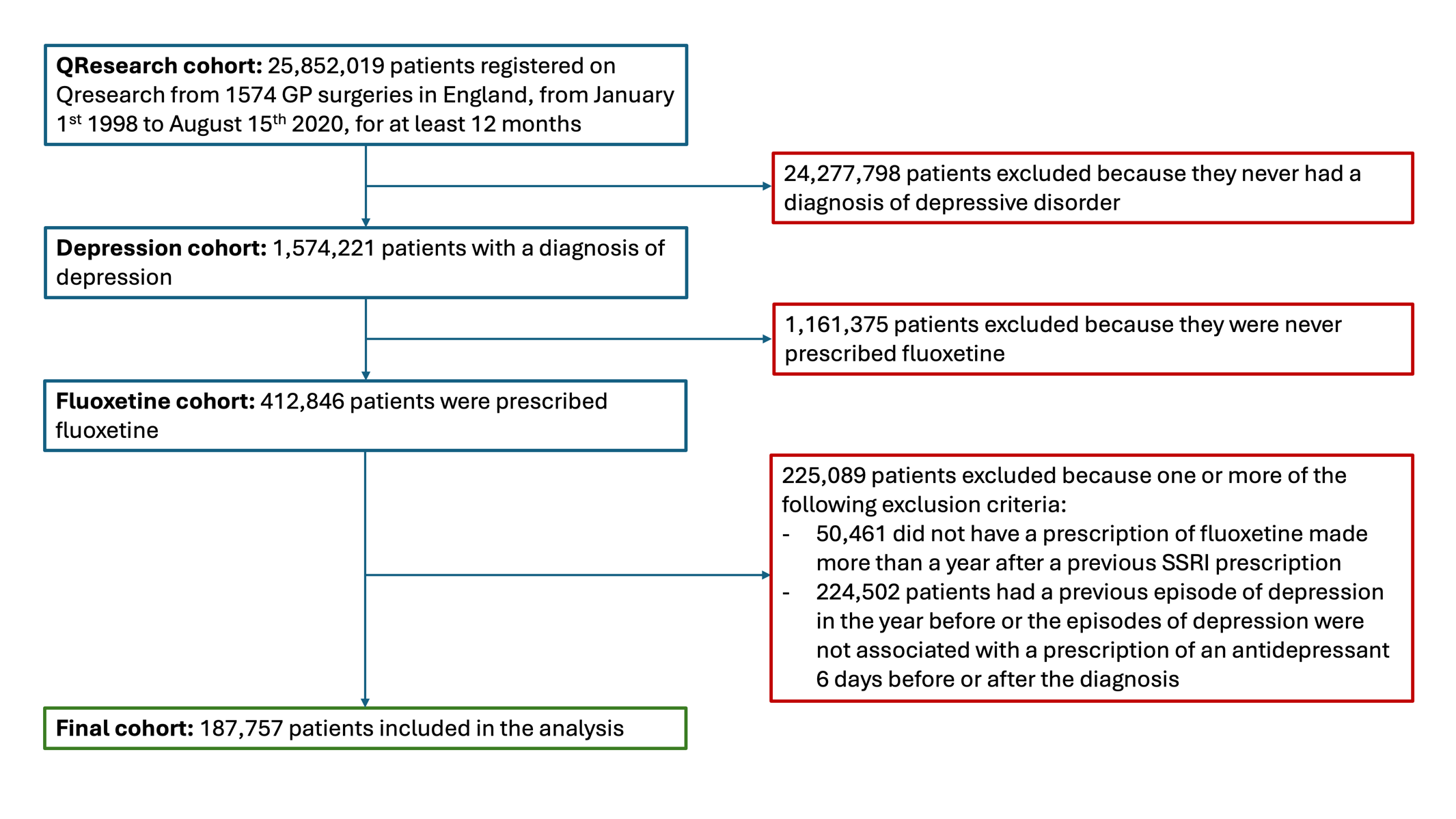


**Supplementary Material Figure 1:** Flow chart of the QResearch cohort (for full information, see ref 20 in the main text)
